# Using Telemedicine During the COVID-19 Pandemic: Attitudes of Adult Health Care Consumers in Israel

**DOI:** 10.3389/fpubh.2021.653553

**Published:** 2021-05-17

**Authors:** Sima Reicher, Tal Sela, Orly Toren

**Affiliations:** ^1^Department of Nursing, Ariel University, Ariel, Israel; ^2^Department of Nursing, Ono Academic College, Kiriat-Ono, Israel; ^3^Department of Behavioral Sciences, School of Social Sciences and Humanities, Kinneret College, Kinneret, Israel; ^4^Hadassah Medical Organization (HMO), Jerusalem, Israel

**Keywords:** telemedicine, attitudes, chronic illness, COVID-19 pandemic, health care policy, adults

## Abstract

**Introduction:** The COVID-19 pandemic has affected health care services worldwide due to lockdowns, prevention measures, and social distancing. During this period, patients, including older adults and those with chronic conditions, need ways to obtain medical attention other than going physically to the clinic, such as telemedicine services. The purpose of the present study was to evaluate attitudes toward telemedicine during the COVID-19 lockdown in Israel, assess willingness to use such services in the future, and evaluate the extent to which consumers have changed their minds regarding these services.

**Method:** A cross-sectional, descriptive, correlational study was conducted among adults (age 20–90) using social media networks (*N* = 693). Data were collected using an online questionnaire explicitly designed to measure attitudes toward telemedicine.

**Results:** Most of the participants had to use telemedicine during the lockdown and were satisfied therewith. The majority also stated that they would continue using telemedicine in the future. However, only a third stated that they had changed their minds regarding telemedicine. The main predictors of willingness to use telemedicine in the future were the necessity of using such services during lockdown, preference for going to a clinic, and satisfaction with telemedicine, alongside gender and having a chronic illness. Importantly, we found that a preference for visiting the clinic was negatively correlated with willingness to use telemedicine in the future. Education and being single were predictors of the change of mind regarding telemedicine. Participants with chronic conditions are more likely to use these services, and specific attention should be directed to their needs. A small portion of the study sample prefers live appointments with a physician.

**Conclusions:** Telemedicine use is rapidly changing. It is vital for health care providers to identify non-telemedicine users and their common characteristics. Monitoring patients' attitudes regarding telemedicine is essential in the future after the pandemic ends. Targeted outreach plans should be formulated. These plans should be directed at identifying barriers to using telemedicine, and they should generate specific, focused plans.

## Introduction

The COVID-19 epidemic has affected millions worldwide, with enormous economic and medical implications. When the WHO Emergency Committee declared COVID-19 a pandemic (1), most countries, including Israel, imposed restrictions intended to address it. These restrictions were gradually increased to a point wherein most countries, including Israel, declared an overall lockdown. The spread of COVID-19 in Israel began at the end of February 2020.

While Israel has an effective health care system, hospitals are overburdened and crowded (2). A state comptroller's report published on March 23, 2020, concluded that the Israeli Ministry of Health (MoH) and the entire hospital system were not fully prepared for a pandemic influenza outbreak (3). This report cites the shortage of hospital beds, isolation rooms, health care workers, and medications, and fully equipped intensive care units.

From the beginning of the outbreak, the Israeli MoH operated on two primary levels: preventing the spread of the virus through the population, and preparing hospitals to treat patients with COVID-19. As a preventative measure, the MoH imposed a policy of quarantining those exposed to the virus, defined as either having been in proximity to a COVID-19 patient, or returning from abroad. The focus was to convert hospital beds (general and intensive care) to treat COVID-19 patients, decrease elective hospitalization, and reduce outpatient volume. Nearly all outpatient care was halted at the community level, then gradually resumed at the beginning of May 2020 (4).

These steps compelled the health care services both in hospitals and in the community to focus almost exclusively on coping with the pandemic. Failing to obtain an adequate response to their medical issues, patients, including the elderly and those suffering from chronic conditions, were often compelled to seek medical treatment in non-traditional ways. Consequently, medical services' quality in the community was affected, prompting patients to look for adequate medical care through digital means. The present study's main goal was to evaluate health care consumers' attitudes regarding telemedicine during the COVID-19 lockdown in Israel.

## Telemedicine

Many countries have begun assimilating remote medicine technologies into their health care services, a trend that was substantially accelerated with the COVID-19 pandemic. The WHO cited digital technology as one of the essential policy services to respond to the COVID-19 emergency. Such services can help caregivers communicate effectively with their patients during the pandemic and provide improved responses to their health concerns. Due to social distancing regulations, telemedicine's extent of care delivery has increased, providing an effective solution for safe communication (5).

Telemedicine enables digital technological improvements to provide lower cost yet still effective ways to extend medical consultation and treatment. Telemedicine's most significant advantages besides cost reduction are increasing medical service availability, enhancing physicians' efficacy, and extending patients' accessibility to care (6, 7). Studies have also advocated telemedicine as a promising solution to improve several chronic medical conditions, including hypertension, obesity, diabetes, depression, and cancer (8).

Telemedicine is defined as using medical data transferred from one source to another via electronic communication to improve clinical health. Telemedicine comprises a growing variety of applications and services such as two-way video, e-mail, smartphone, and other telecommunication technologies (9). These technologies enable communication between geographically remote patients and caregivers to provide care, consultation, follow-up, guidance and health education, medical intervention, monitoring, and remote hospitalization. They also enable inexpensive and effective ways to obtain medical care and overcome geographical distance (6), thereby reducing emergency room visits and hospitalization rates (10)_._

Digital caregiver–patient communication is divided into synchronous and asynchronous communication. The Office of the National Coordinator for Health Information Technology (ONC) defines synchronous telemedicine as “live video-conferencing,” which is an “interactive video connection that transmits information in both directions during the same period“ [American Telemedicine Association—ATA; (11)]. The synchronous method's major advantage is the efficacies gained by eliciting relevant details during the session by seeking additional information or data, and in many cases providing a clinical diagnosis or advice within the session. The ATA defines asynchronous telemedicine as “store-and-forward transmission of medical images and/or data because the data transfer takes place over a period of time, and typically in separate time frames. The transmission typically does not take place simultaneously.” This type of telemedicine is provided via a virtual clinic to which the patient does not need to come physically, but maintains communication with the health care provider in her own time through phone calls, video calls, and written correspondence.

Data from the US Department of Health show that the number of patients with government health insurance who used telemedicine rose from 11,000 users on March 7, 2020, to 1.3 million as of April 18, 2020 (5). According to Global Market Insights, Inc., telemedicine's market size is forecasted to exceed $175 billion by 2026, and the increasing prevalence of chronic diseases across the globe will bolster the demand therefor (12).

Following the policy of the Digital Israel project (13), the Israeli MoH has declared as its mission “to bring about a leap in the health system that will enable it to become sustainable, advanced, innovative, renewed, and constantly improving, by optimally leveraging the information, and communication technologies available to the entire Israeli population.” In other words, the global acceleration in technological development and the digital revolution creates an opportunity to implement further and develop diverse telemedicine options.

Studies show that interventions based on telemedicine may yield results similar to live appointments. For example, an Israeli study examined the effectiveness of reducing patients' sense of distress by using telemedicine. The study compared telephone, video, and face-to-face conversations between caregivers and patients. The findings suggest similar effects in reducing distress between face-to-face appointments and telemedicine (6). A literature review on the current state of telehealth shows that, generally, in most cases, telehealth appears to be equivalent to face-to-face care or, in some cases, has better outcomes than do services such as mental assessment and treatment, rehabilitation consultation, anti-coagulation management, and older adults' nutrition management (14).

Edwards et al. (15) showed that patients with chronic diseases are interested in using telemedicine, regardless of their health status and age. A study conducted in Italy's Veneto region revealed that chronic patients reported high satisfaction with telemedicine services in the short term and even higher satisfaction after 1 year. These findings indicate that the use of telemedicine likely requires an adjustment period. Interestingly, although patients have expressed high satisfaction with these services, they did not perceive telemedicine as a substitute for traditional medical care, but rather only as a supplement to help them manage their condition (16).

Studies on telemedicine use have focused on various adult age groups [18 and above; (7, 8, 17)] and found that telemedicine use varies by patient age. For example, Jaffe et al. (17) found that telemedicine use in the 18–44 age group was significantly higher than in the 45–64 age group and higher than adults aged 65 and over. Kruse et al. (7) reported that age-related barriers exist due to lack of exposure to the new technology and patients' lack of training, and claimed that the technology acceptance gap among older patients is consistent with the patients' preferences for face-to-face care.

An Israeli study conducted in 2017 (18) examined the use of telemedicine among adult members of a leading Health Maintenance Organization (HMO), Maccabi Healthcare Services. The study found that telemedicine use was negatively correlated with age: 69% of participants aged 45–54 reported using online services, and about 60–63% of participants aged 55–74 reported using these services. However, in the oldest age group (75 and above), only 43% reported using these services. Overall, around 60% of the study participants (*N* = 331) reported having used telemedicine. Jaffe et al. (16) noted that digital technology access and use has increased dramatically in the last decade among older adults. Therefore, despite the differences in the usage of various age groups, the fact that digitalization is becoming a part of regular life may eliminate the barrier to telemedicine use.

Another factor that may affect telemedicine use is gender, although findings regarding usage differences are mixed. For example, Hilbert suggested that women tend to be latecomers regarding digital applications (19). Hargittai and Shafer tested how gender and self-perceived abilities are related to online abilities (20). They found that women's lower self-assessment regarding their online skills affects the extent of their online behavior and the types of technology used. Guo et al. examined mobile-health use in China (21). They found that threat appraisal (i.e., how one assesses the severity of the situation) was more related to gender and age, whereas coping appraisal (i.e., how one responds to the situation) was better among men and youths. A recent cohort study in the US examined telemedicine vs. in-person encounters between March 2019 and March 2020. In this study, no difference was observed for gender, ethnicity, socioeconomic status, or health behavior (17).

The COVID-19 pandemic is a resounding reminder that chronic and adult patients need to be treated anywhere, anytime, taking into account the new restrictions, regulations, and changes in the consumption of health care services (12). As telemedicine was available to some extent in Israel pre-pandemic, the question is whether chronic and adult health care consumers are ready or willing to use this technology for any health care need. This question is crucial considering the trends of the Israeli MoH policy regarding the implementation of telemedicine as well as patient-centered care and personalized medicine in the national plan for digital health (22).

Given these trends, this study's main goal was to explore attitudes toward using telemedicine during Israel's COVID-19 lockdown. Specifically, we examined attitudes regarding the necessity of using telemedicine during the crisis, patients' preference for going to a clinic, and how satisfied patients were with the provided telemedicine services. We also aimed to assess the influence of attitudes toward telemedicine on willingness to use such services in the future and to evaluate to what extent patients have changed their attitudes toward telemedicine.

## Methods

### Design and Data Collection

The study was a cross-sectional, descriptive, correlational study conducted as part of a more extensive study to build and validate a new, general-purpose questionnaire (not COVID-19 related) to measure attitudes toward telemedicine and map possible barriers that patients may face to using such services.

After obtaining institutional ethical approval, a pilot study was conducted to evaluate the questionnaire's reliability and validity. Data were collected online between April 21 and May 16, 2020. During this time, regulations in Israel changed from complete lockdown to activity restrictions (23). These restrictions included opening commercial services and industries under certain conditions (health declarations, body temperature measurement, social distancing, etc.). The number of people allowed in closed areas was limited. Quarantine was mandatory for those returning from abroad or exposed to a verified COVID-19 patient. During this time, a sharp decrease in hospitalization was recorded. On April 30, Israel adopted the WHO recommendations for considerations in adjusting public health and social measures in the context of COVID-19 (24).

Participants in this study were recruited through ads on websites aimed at general social media users, the elderly population, and patients with chronic diseases [Motke, an online portal and unique social network platform aiming to support Israel's elderly population (25), and the Camoni portal, a social media site focusing on chronic health conditions (26)]. We also used chain referral sampling through social networks such as Facebook and WhatsApp. Of 944 participants who followed the questionnaire's link, 933 signed the consent form and agreed to participate in the research. Of those, 693 fully completed the survey. Table 1 presents descriptive statistics of the sample.

**Table 1:** Descriptive statistics of the sample (*N* = 693).

**Variable**	**Status**	**Frequency**	**%**
Gender	Female	398	57.4
	Male	295	42.6
Education level	Academic education	408	58.9
	High school/vocational education	229	33.0
	Other education	56	8.1
Marital status	Partnered	412	59.5
	Single	67	9.7
	Divorced	124	17.9
	Widowed	72	10.4
	Other	18	2.6
Economic status	Poor	38	5.5
	Reasonable	281	40.5
	Good	314	45.3
	Excellent	60	8.7
Chronic illness	No (none diagnosed)	239	34.5
	Yes (one or more diagnosed)	454	65.5
Age	Mean (SD)	64.21	(12.89)
	Median (IQR)	67	(12.00)
	Minimum	20	
	Maximum	90	
	Skewness (SD)	−1.30	(0.09)
	Kurtosis (SD)	1.75	(0.18)
**Age bracket**	***N***	**Mean (SD)**	**Median (IQR)**
Below 40	50	30.78 (6.31)	31.5 (9)
40–49	42	45.55 (2.50)	45 (4)
50–59	74	55.61 (2.62)	56 (5)
60–69	301	66.00 (2.95)	66 (5)
70 and above	226	75.52 (4.14)	74 (6)

*Total number of participants N = 693*.

### Research Tool

A questionnaire was designed to assess 'participants' attitudes toward telemedicine services during the COVID-19 lockdown. The questionnaire includes five statements on a five-point Likert scale (1—“Strongly disagree” to 5—“Strongly agree”). Participants were asked to express the extent of their dis/agreement with each of the following statements:

1. **Item 1**: *Necessity* of using telemedicine—“Being isolated during the COVID-19 crisis required me to use telemedicine to receive health care/counseling.”
2. **Item 2**: *Preference* for going to a clinic—“Despite the availability of telemedicine during the COVID-19 lockdown, I preferred going to the clinic.”
3. **Item 3**: *Satisfaction* with telemedicine services—“In general, during the COVID-19 period, I am satisfied with the medical services provided through digital technology.”
4. **Item 4**: *Willingness* to use telemedicine in the future—“I will continue to use telemedicine even after the COVID-19 pandemic ends.”
5. **Item 5**: *Change of mind* about telemedicine—“Being isolated during the COVID-19 crisis has changed my mind about using telemedicine for my health needs.”

In addition, a socio-demographic section was included, collecting age, gender, socioeconomic status, education, occupation, and the presence of chronic conditions. Descriptive statistics are presented in Table 1.

### Statistical Analysis

First, we explored our results using descriptive analysis. Next, we calculated Spearman's rank correlations for the various telemedicine items. Then, we fitted ordered logistic regression (OLR) models to our dependent variables that included one of two items: *Willingness to use* telemedicine in the future (Item #4) and *Change of mind* regarding telemedicine (Item #5). We trichotomized these five-point Likert scales. For each item, we produced a three-point scale by collapsing responses 1 and 2 (“Strongly Disagree” and “Disagree,” respectively) into one category and responses 4 and 5 (“Agree” and “Strongly agree”, respectively) into another category, yielding an ordinal scale of 3 levels: 0 = “Disagree,” 1 = “Neutral,” and 2 = “Agree.”

Independent variables included socio-demographic (gender, chronic illness, age bracket, personal status, economic status, and education level) alongside the three items from the questionnaire (necessity of using telemedicine, preference for going to a clinic, and satisfaction with telemedicine services; all entered as categorical variables, using Response 1—“Strongly disagree” as the baseline level).

For each dependent variable, we fitted several models. First, we examined the relationship between background variables and the dependent variable. Next, we added the telemedicine items. Finally, we used a forward-stepwise method as a variable selection method. We used various evaluation matrices to test model fit (e.g., Bayesian Information Criterion—BIC; McFadden Pseudo *R*^2^). The Likelihood-ratio test (LR test) was used to test nested models. The Brant test and other regression diagnostics were used to evaluate model assumption. We present Average Adjusted Predictions (AAPs) and Adjusted Predictions at Representative values (APRs) for both dependent variables for ease of interpretation. All regression analyses employed robust cluster errors. The statistical significance was set at a *p*-value of 0.05. When needed, FDR correction was applied to address multiple comparisons. Data were analyzed using SPSS v.25 & Stata v.16.

## Results

Sixty-four percent of the participants agreed or strongly agreed that they had had to use telemedicine during the COVID-19 lockdown (Table 2). Around the same frequency (~63%) disagreed or strongly disagreed with preferring to go to the clinic during the lockdown. Also, the same proportion of participants were satisfied with telemedicine services during the COVID-19 lockdown. Most of the participants (~77%) agreed or strongly agreed that they would continue to use telemedicine in the future. However, only 31% agreed or strongly agreed that they had changed their minds regarding these services.

**Table 2:** Participants' responses (%) to the five-item questionnaire (*N* = 693).

	**(1) Strongly disagree**	**(2) Disagree**	**(3) Undecided**	**(4) Agree**	**(5) Strongly agree**
(1) Item 1: Necessity of using telemedicine during the COVID-19 crisis	12.1	14.2	9.5	36.4	27.8
(2) Item 2: Preference for going to a clinic during the COVID-19 crisis	36.7	27.1	10.6	16.8	8.8
(3) Item 3: Satisfaction with telemedicine services during the COVID-19 crisis	3.6	11.5	20.3	42.7	21.9
(4) Item 4: Willingness to use telemedicine in the future	3.9	4.9	14.0	44.3	32.9
(5) Item 5: Change of mind regarding telemedicine	22.9	28.9	17.0	23.0	8.2

Table 3 presents the correlations between the various items. We found that the necessity of using telemedicine during the COVID-19 lockdown was positively correlated with both willing to use telemedicine in the future and with change of mind regarding telemedicine. That is, participants who agreed more strongly that they had had to use or receive telemedicine services during the COVID-19 lockdown were those who were more disposed to continue using these types of services in the future and are possibly more likely to change their minds regarding their use of this type of services.

**Table 3:** Spearman's rank correlation between questionnaire items (*N* = 693).

	**(1)**	**(2)**	**(3)**	**(4)**	**(5)**
(1) Item 1 Necessity of using telemedicine during the COVID-19 crisis	1				
(2) Item 2 Preference for going to a clinic during the COVID-19 crisis	−0.198^**^	1			
(3) Item 3 Satisfaction with telemedicine services during the COVID-19 crisis	0.352^**^	−0.202^**^	1		
(4) Item 4 Willingness to use telemedicine in the future	0.320^**^	−0.404^**^	0.451^**^	1	
(5) Item 5 Change of mind regarding telemedicine	0.218^**^	0.062	0.060	−0.029	1

** *p <0.001*.

Preference for going to a clinic during the COVID-19 lockdown was negatively correlated with willingness to use telemedicine in the future. That is, participants who preferred to go to the clinic during the COVID-19 lockdown were less likely to state that they would agree to continue using telemedicine in the future. Moreover, a positive correlation was found between participants' satisfaction with telemedicine services and their willingness to continue to use these services. Participants who reported a higher level of satisfaction with telemedicine services tended to state that they would agree to use telemedicine in the future.

Overall, the pattern of results that emerges from the correlation analysis points to only one item—necessity of use—being correlated with change of mind regarding telemedicine. However, necessity of use, preference for going to a clinic, and satisfaction with telemedicine services were correlated with willingness to use telemedicine in the future. Also, a preference for visiting the clinic was negatively correlated with willingness to use telemedicine in the future. Finally, the results' patterns were similar in a subpopulation analysis, wherein we restricted the age range to only those aged 60 and above (*N* = 527) or to only patients with chronic illness (*N* = 454) (see Supplementary Tables 1–4 in Supplementary Material). That is, the correlations between the various items are stable.

### Ordered Logistic Regression Models

#### Predicting Willingness to Use Telemedicine in the Future

We fitted various OLR models (as elaborated upon in Supplementary Material), and we present the best-fitted model for predicting willingness to use telemedicine in the future. As shown in Table 4, we found that gender, chronic illness, and Items 1–3 were associated with willingness to use telemedicine in the future. Specifically, most participants (77%) agreed that they would continue to use telemedicine in the future, nearly 14% are undecided, and nearly 9% disagreed with this statement. As presented in Table 4, we found a main effect for gender (OR = 2.16, 95% CI: 1.40, 3.32): Around 82% of the male participants agreed that they would continue to use telemedicine in the future; 11% were undecided, while 6% disagreed with this statement. For women, around 73% agreed that they would continue to use telemedicine in the future, 16% were undecided, and nearly 11% disagreed with this statement. In summary, while only 17% of males disagreed or did not strongly agree that they would continue to use telemedicine, nearly 27% of the women in the sample disagreed or did not strongly agree to use telemedicine in the future.

**Table 4:** Results of OLR model predicting willingness to use telemedicine in the future (0 = “Disagree,” 1 = “Neutral,” and 2 = “Agree”) (*N* = 693).

	**OR**	**SE^#^**	***z***	***P* > z**	**95% CI**	
Gender (male = 1)	2.16	0.47	3.5	0	1.4	3.32
Chronic illness (yes = 1)	1.58	0.33	2.17	0.03	1.05	2.39
**Item 1^∧^**						
2–“Disagree”	1.16	0.37	0.46	0.65	0.62	2.16
3–“Undecided”	1.57	0.58	1.23	0.22	0.76	3.25
4–“Agree”	2.19	0.68	2.53	0.01	1.19	4.01
5–“Strongly agree”	3.79	1.41	3.59	0	1.83	7.86
**Item 2^∧^**						
2–“Disagree”	0.55	0.16	−2.05	0.04	0.31	0.97
3–“Undecided”	0.4	0.14	−2.69	0.01	0.21	0.78
4–“Agree”	0.17	0.05	−5.58	0	0.09	0.32
5–“Strongly agree”	0.09	0.03	−6.71	0	0.04	0.18
**Item 3^∧^**						
2–“Disagree”	2.25	1.09	1.66	0.1	0.86	5.84
3–“Undecided”	1.73	0.83	1.15	0.25	0.68	4.42
4–“Agree”	6.76	3.25	3.98	0	2.64	17.35
5–“Strongly agree”	14.37	8.85	4.33	0	4.3	48.03
Cut point 1	−1.02	0.54			−2.08	0.04
Cut point 2	0.45	0.54			−0.61	1.5
**Fit indices**						
AIC	784.25					
BIC	856.91					
McFadden pseudo *R*^2^	0.21					
Nagelkerke pseudo *R*^2^	0.34					
Model df	14					

*OR, Odds Ratio; SE*

# *, Robust standard errors*;

∧ *, for items 1 to 3, response no. 1, “Strongly Disagree” serves as the baseline category; AIC, Akaike Information Criterion; BIC, Bayesian Information Criterion*.

We also found a main effect for chronic illness (OR = 1.58, 95% CI: 1.05, 2.39): Around 80% of participants with any type of chronic illness agreed that they would continue to use telemedicine in the future; 13% were undecided, and 7% disagreed with this statement. For participants without chronic illness, around 73% agreed that they would continue to use telemedicine in the future, 16% were undecided, and 11% disagreed with this statement. In summary, while only 20% of participants with chronic illness disagreed or did not fully agree that they would continue to use telemedicine service in the future, 27% of non-chronic participants in the sample disagreed or did not fully agree to use telemedicine in the future.

Regarding both gender and chronic illness variables, the APRs for a man with a chronic illness agreeing to use telemedicine in the future were around 84.5% and those for a man without chronic illness were 75.5%, while the APRs for a woman with chronic illness agreeing to use telemedicine in the future was around 79.5% and those for a woman without chronic illness 69%.

**Figure 1:**
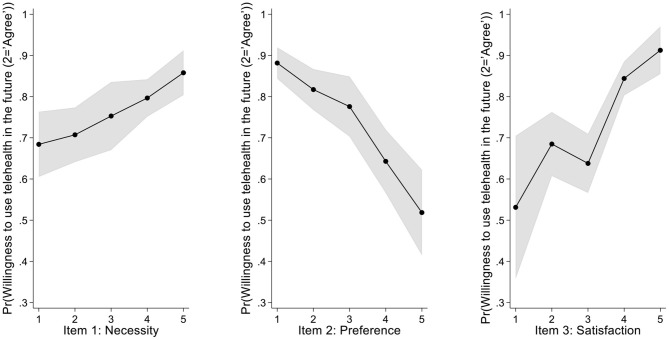
Average Adjusted Predictions for Items 1 to 3. The *y*-axis represents the likelihood of agreeing with the statement “I will continue to use telemedicine in the future”; shaded gray represents 95% CI. *Post-hoc* analysis reveals a linear trend relating to the correlation between a given item and the likelihood of agreeing with the statement. Specifically, we found a linear trend for Item 1 ($\chi_{1}^{2}$ = 16.54, *p* < 0.001; contrast = 0.060; 95% CI: 0.031, 0.089), for Item 2 ($\chi_{1}^{2}$ = 44.36, *p* < 0.001; contrast = −0.113; 95% CI: −0.142, −0.084), and for Item 3 ($\chi_{1}^{2}$ = 16.54, *p* < 0.001; contrast = 0.104; 95% CI: 0.073, 0.134). Consistent with the correlation analysis, we found an association between each item and the intent to use telemedicine in the future.

Finally, as shown in Table 4 and Figure 1, similar to the correlation analysis, all three items were significantly associated with willingness to use telemedicine in the future.

#### Predicting Change of Mind Regarding Telemedicine

We fitted various OLR models (as elaborated upon in Supplementary Material), and we present the best-fitted model for predicting change of mind regarding telemedicine. More than half of the participants (52%) disagreed that they had changed their mind toward telemedicine during the COVID-19 lockdown, nearly 17% were undecided, and nearly 32% agreed that they had changed their minds. As shown in Table 5, education level and being single are correlated with change of mind. Specifically, we found that high school/vocational education participants significantly differed from those with academic education (OR = 1.69, 95% CI 1.22, 2.34): While only 27% of participants with academic education agreed that they had changed their minds regarding telemedicine, around 37% of those with high school/vocational education reported that they had changed their minds. We also found that single participants differ from partnered ones (OR = 0.47, 95% CI 0.27, 0.79), i.e., nearly 20% of the single participants reported having changed their minds, while 34% of partnered participants reported having changed their minds regarding telemedicine.

**Table 5:** Results of OLR model predicting change of mind regarding telemedicine (0 = “Disagree,” 1 = “Neutral,” and 2 = “Agree”) (*N* = 693).

	**OR**	**SE^#^**	***z***	***P* > *z***	**95% CI**	
**Personal status (partnered as baseline)**						
Single	0.467	0.127	−2.810	0.005	0.274	0.795
Divorced	0.749	0.160	−1.350	0.175	0.493	1.138
Widowed	0.901	0.218	–.430	0.665	0.560	1.448
Other	0.829	0.414	−0.380	0.707	0.311	2.207
**Education level (academic education as baseline)**						
High school/vocational education	1.692	0.281	3.160	0.002	1.221	2.344
Other	1.322	0.356	1.040	0.299	0.781	2.240
**Item 1^∧^**						
2–“Disagree”	0.897	0.295	−0.330	0.741	0.471	1.709
3–“Undecided”	1.587	0.482	1.520	0.128	0.875	2.879
4–“Agree”	3.154	0.857	4.230	0.000	1.852	5.373
5–“Strongly agree”	3.493	1.059	4.120	0.000	1.928	6.329
**Item 2^∧^**						
2–“Disagree”	1.126	0.231	0.580	0.563	0.753	1.684
3–“Undecided”	1.931	0.474	2.680	0.007	1.194	3.123
4–“Agree”	1.588	0.375	1.950	0.051	0.999	2.524
5–“Strongly agree”	1.740	0.488	1.980	0.048	1.005	3.013
**Item 3^∧^**						
2–“Disagree”	1.909	0.792	1.560	0.119	0.846	4.306
3–“Undecided”	3.295	1.311	3.000	0.003	1.511	7.185
4–“Agree”	2.014	0.787	1.790	0.073	0.936	4.332
5–“Strongly agree”	2.459	1.034	2.140	0.032	1.079	5.607
Cut point 1	1.977	0.463			1.069	2.885
Cut point 2	2.771	0.465			1.860	3.682
**Fit indices**					
AIC	1,353.771					
BIC	1,444.592					
McFadden Pseudo *R*^2^	0.057					
Nagelkerke Pseudo *R*^2^	0.126					
Model df	20					

*OR, Odds Ratio; SE*

# *, Robust standard errors*;

∧ *, for Items 1 to 3, response no. 1, Strongly Disagree” serves as the baseline category; AIC, Akaike Information Criterion; BIC, Bayesian Information Criterion*.

Finally, consistent with the correlation analysis, *post-hoc* analyses (using FDR correction) revealed a linear trend for Item 1 only ($\chi_{1}^{2}$ = 39.90, *p* <0.001; contrast = 0.097; 95% CI: 0.067, 0.127); i.e., we found a positive correlation between the necessity of using telemedicine during the COVID-19 crisis and having changed one's mind regarding telemedicine.

## Discussion

In Israel, as in other developed nations, the COVID-19 pandemic has accelerated the use of telemedicine. The current study examined the attitudes of adults toward the use of telemedicine during the COVID-19 lockdown. The results show that most participants preferred to use telemedicine and were satisfied with its use during this period. The health system needs to provide virtual medical care whenever possible to keep patients at home while still offering them access to necessary medical care (27). Various studies and surveys conducted during the lockdown found high satisfaction with the telecare provided (28). Our findings are consistent with these reports (18, 19), showing high satisfaction with telemedicine services.

In the current study, nearly 80% of the participants are willing to use telemedicine in 2020 and the future. We can assume that this finding is associated with an increase in the tendency to use these services, based on data from one HMO, in which 60% of the members surveyed reported having used them in the past (18). This trend is consistent with many countries reporting an increase in telemedicine use during lockdowns. This finding should be treated with caution, as other factors may have contributed to this phenomenon, such as technological progress and HMOs' efforts to encourage patients to use these services.

Our results suggest that most participants disagreed with going to the clinic during the outbreak, and those who did so found themselves using telemedicine to obtain medical treatment. Similarly, based on a survey of 2,700 patients in the US, Heat (28) stated that 4 in 10 patients began using a new app or digital technology to stay connected to their health care providers at the onset of the COVID-19 epidemic. Previous studies conducted in Israel showed that patients and physicians are willing to use digital technology instead of face-to-face appointments when their preferences are considered (29). Another study found that the high use of HMOs' mobile health apps across the socio-demographic spectrum indicates telemedicine's high perceived usefulness (30). The lockdown prompted many clinicians and patients to realize these tools' potential and compelled them—some for the first time—to utilize them when face-to-face appointments were precluded (31).

In the present study, participants rated their degree of willingness to use telemedicine in the future. Studies have found strong correlations between willingness, intent, and behavioral expectations (32). Our findings show that gender and chronic illness are correlated with participants' willingness to use telemedicine in the future. Specifically, around 82% of males and 73% of females agreed that they would continue to use telemedicine in the future. Our results also showed a correlation between chronic illness and willingness to use telemedicine in the future. Around 80% of chronically ill participants agreed that they would continue to use telemedicine in the future vs. 73% of those not chronically ill, which is consistent with Edwards's et al. (15) findings.

The necessity of using telemedicine and satisfaction therewith positively correlates with willingness to use them in the future, while going to a clinic was negatively correlated with this intention. One possible explanation for these results stems from the premise of planned behavior theory (33), according to which positive or negative attitudes toward a given action can predict one's intention and behavior. Accordingly, during the outbreak, some of the participants were already familiar with telemedicine. As a result, they expressed a high level of satisfaction with and willingness to use telemedicine in the future. The same reasoning can be applied to those participants who preferred to go to a clinic: Despite the lockdown, these participants were entrenched in their habit, were less satisfied with telemedicine services, and accordingly expressed no intention of using them in the future. However, this explanation cannot account for the possibility that some participants were compelled to use telemedicine during the outbreak and the positive correlation between the necessity of using such services and willingness to use them in the future.

In contrast to the attitude influence behavior framework, other accounts such as cognitive dissonance theory (34) and self-perception theory (35) have claimed that behavior can shape attitude in many situations. People have a strong need to maintain cognitive consistency (36), tend to act in a manner that is consistent with previous actions or behavior (37), and it has been found that past behavior can be a predictor of future behavior (38). Before the COVID-19 pandemic, telemedicine was often used as a secondary alternative to visiting a clinic. The lockdown rapidly changed the picture, as most participants were compelled to use telemedicine as a nearly exclusive alternative. We propose that this impelled behavioral change triggered a change in participants' attitudes toward telemedicine, thus increasing their willingness to use it in the future. The challenge, of course, is to continue the trend and establish the use of telemedicine as an equivalent alternative to face-to-face treatment in the clinical setting.

In the present study, we also found that only 32% of the participants agreed that telemedicine had changed their minds. One possible explanation for that may lie in the study sample: Some study participants are members of social network communities that include support groups and chronic patients. Therefore, it can be assumed that those participants are accustomed to using digital technology, have already formed their habits and opinions regarding telemedicine, and feel no need to change their intentions to use it. Likewise, in several studies, habits have been found to moderate the relationship between intentions and behavior (39). For example, behaviors that are engaged infrequently in stable contexts support the development of habits, and thus the impact of intention on behavior is attenuated (38).

Another central theme related to telemedicine use surrounded the patient–physician relationship. Several studies have linked participants' preferences for telemedicine to their familiarity with the physician. For example, in a study in South Carolina, patients expressed the importance of the patient–physician relationship, and it was the leading factor in choosing the type of service, i.e., face-to-face vs. telemedicine (40). However, Valikodath et al. found that among diabetic patients, only those who were unreceptive to telemedicine strongly valued their patient–physician relationship (41).

“No man is an island,” as John Donne elegantly put it. Humans need to establish an emotional bond with a caregiver and want to be part of a relationship, while telemedicine may be perceived as indifferent to them, and thus they would avoid using it. Härtel and Russell-Bennett defined emotional loyalty as the psychological preference for a brand that consists of positive feelings and affective attachment that facilitates its purchase or use in the future (42). Such emotional loyalty is crucial to health care consumers; thus, policymakers need to be aware of how well-telemedicine meets the consumers' specific needs, emphasizing personal relationships. Consumers who still prefer a face-to-face meeting with the physician should not be ignored. The health care system should be prepared to meet these patients' needs, certainly in a pandemic or other national emergency.

Personal status and education were found to correlate with changing of mind. Being single decreased the probability of changing one's mind regarding telemedicine, while high school education increased it. A possible explanation for these findings could stem from the frequency with which digital technology is used. That is, the more the individual is accustomed to using telemedicine, the less likely he/she will be to change his/her mind regarding it. As the frequency of telemedicine use among those with a high school education is lower than for those with post-secondary education, the likelihood of the latter changing their minds is higher.

The current study has several limitations. Firstly, this study is a cross-sectional one based on a convenience sample and includes mainly older adults. Despite a large number of participants, the sample was not representative and was left-skewed in terms of participant age. Moreover, a sizable portion of the study sample was drawn from a digital list of people with e-mail addresses. It is possible that sample participants, having used at least some digital technology, were more likely to use other digital technology related to health. That is, digital recruitment may be one reason for the high number of telemedicine users among the current study participants. Using other sampling methodologies (postal or telephone survey) could have examined people's attitudes toward telemedicine among those who do not use the internet. Nonetheless, we note that although the sample is not representative, the results remained stable when we ran all the analyses on chronic patients only.

Secondly, patients' characteristic measures (other than demographics) were not part of the study. These variables should be incorporated into future research to better explain and understand the outcomes. Finally, the study employs single items to measure attitudes toward various aspects of telemedicine use. Single items may suffer from accuracy, content validity, and reliability problems (in particular, their internal consistency cannot be estimated). Nonetheless, many studies in various fields have been using a single-item scale to measure various constructs when time is constrained to obtain preliminary yet critical insight into important health and social phenomena.

## Conclusions

The pandemic outbreak has created an opportunity at the state and institutional levels to promote telemedicine more vigorously and develop various services faster than planned. This rapid acceleration of telemedicine accords with the Israeli policy of promoting digital services and making them sustainable, advanced, innovative, and continuously improving. Although most participants were satisfied with current telemedicine and willing to use it in the future, monitoring patients' attitudes regarding telemedicine after the pandemic is essential. As a small yet still significant portion of participants prefers a live meeting with a physician, it is also essential for HMOs to facilitate specific encounters based on this expectation, alongside identifying barriers to patients' using telemedicine. As telemedicine's use is dramatically increasing, there is a need to disseminate information about each service's availability and its use, and its advantages over face-to-face appointments. Further research is needed on this topic to guide policymakers in formulating strategies that promote telemedicine implementation while addressing possible gender gaps therein, and to examine the study variables' causal influence on adopting telemedicine services.

## Implications

It is essential to distinguish between groups of chronic patients and target telemedicine services accordingly. The HMOs should create targeted interventions to identify patients' preferences and maintain the patient–health care provider relationship for any given group of patients. Additionally, HMOs need to identify specific barriers and group characteristics to using telemedicine and, based thereon, to formulate targeted outreach plans. These plans should be directed at overcoming telemedicine barriers, such as initiating scheduled home visits and scheduled telephone inquiries for regular health assessment.

Plans should also include providing any health assistance needed (transportation to the clinic, home laboratory services, delivery of medicines, etc.). Policymakers need to address the extent to which consumers would enjoy the quality of care delivered via telemedicine and prefer it even post-COVID. As most countries have limited resources due to increased life expectancy and more chronic patients, it is incumbent upon policymakers and health care providers to learn about future consumer behavior.

## Data Availability Statement

The raw data supporting the conclusions of this article will be made available by the authors, without undue reservation.

## Ethics Statement

The studies involving human participants were reviewed and approved by Ethical approval was granted from the Ethical Committee at Ono Academic College. Written informed consent for participation was not required for this study in accordance with the national legislation and the institutional requirements.

## Author Contributions

SR and OT: major contributor in designing the study, data collection, data interpretation, and writing the manuscript. TS: major contributor to data analysis, data interpretation, and writing the manuscript. All authors contributed to the article and approved the submitted version.

## Conflict of Interest

The authors declare that the research was conducted in the absence of any commercial or financial relationships that could be construed as a potential conflict of interest.

## Abbreviations

MoH: Israeli Ministry of Health
WHO: World Health Organization
ATA: American Telemedicine Association
HMO: Health Maintenance Organization
ONC: Office of the National Coordinator for Health Information Technology.
