# Supplementary material for: Using Telemedicine During the COVID-19 Pandemic: Attitudes of Adult Health Care Consumers in Israel
Source: Front Public Health. 2021 May 17;9:653553. doi: 10.3389/fpubh.2021.653553 (PMC8165259; doi:10.3389/fpubh.2021.653553)
Supplement: Supplementary file 1 [file Table_1.DOCX]

**Supplementary material – Results section**

**Subpopulation analysis**

As mentioned in the main text, we also examined if the pattern of the results we found based on the whole sample (N=693) is similar if we look at specific subpopulations in the sample, such as adults aged 60 and above and patients who suffer from chronic illness. When we restrict the age range to include only people aged 60 and above (N=527), the results' pattern is similar to the whole sample in terms of participants' responses (%) to the 5-item telemedicine questionnaire (Table S1) and the correlation between the items (Table S2).

In the same vein, when we include only people with chronic illness (N=454), the results' pattern is similar to the whole sample (see Tables S3 & S4).

**Table S1.** **Participants' responses (%) to the 5-item questionnaire - age 60 and above** **(N = 527)**

|  | **(1)**  **Strongly disagree** | **(2)**  **Disagree** | **(3)**  **Undecided** | **(4)**  **Agree** | **(5)**  **Strongly agree** |
| --- | --- | --- | --- | --- | --- |
| **(1)** **Item 1:** Necessity of using telemedicine during the COVID-19 crisis | 12.1 | 14.0 | 10.1 | 36.8 | 26.9 |
| **(2)** **Item 2:** Preference for going to a clinic during the COVID-19 crisis | 37.4 | 27.7 | 8.9 | 16.1 | 9.9 |
| **(3)** **Item 3:** Satisfaction with telemedicine services during the COVID-19 crisis | 3.8 | 11.2 | 20.9 | 43.6 | 20.5 |
| **(4)** **Item 4:** Willingness to use telemedicine in the future | 4.7 | 3.8 | 14.0 | 45.7 | 31.7 |
| **(5)** **Item 5:** Change of mind regarding telemedicine | 22.0 | 27.3 | 17.6 | 25.0 | 8.0 |

**Table S2. Spearman's rank correlation between questionnaire items (Age 60 and above; N = 527)**

|  | **(1)** | **(2)** | **(3)** | **(4)** | **(5)** |
| --- | --- | --- | --- | --- | --- |
| **(1)** **Item 1:** Necessity of using telemedicine during the COVID-19 crisis | 1 |  |  |  |  |
| **(2)** **Item 2:** Preference for going to a clinic during the COVID-19 crisis | -0.178** | 1 |  |  |  |
| **(3)** **Item 3:** Satisfaction with telemedicine services during the COVID-19 crisis | 0.368** | -0.201** | 1 |  |  |
| **(4)** **Item 4:** Willingness to use telemedicine in the future | 0.310** | -0.370** | 0.464** | 1 |  |
| **(5)** **Item 5:** Change of mind regarding telemedicine | 0.259** | 0.048 | 0.099* | 0.033 | 1 |

*Note:* *p < 0.05, **p < 0.001

**Table S3. Participants' responses (%) to the 5-item questionnaire; only patients with chronic illness** **(N = 454)**

|  | **(1)**  **Strongly disagree** | **(2)**  **Disagree** | **(3)**  **Undecided** | **(4)**  **Agree** | **(5)**  **Strongly agree** |
| --- | --- | --- | --- | --- | --- |
| **(1)** **Item 1:** Necessity of using telemedicine during the COVID-19 crisis | 10.8 | 13.9 | 9.3 | 36.3 | 29.7 |
| **(2)** **Item 2:** Preference for going to a clinic during the COVID-19 crisis | 35.5 | 27.5 | 10.1 | 17.4 | 9.5 |
| **(3)** **Item 3:** Satisfaction with telemedicine services during the COVID-19 crisis | 4.0 | 10.8 | 16.3 | 45.4 | 23.6 |
| **(4)** **Item 4:** Willingness to use telemedicine in the future | 4.0 | 3.3 | 11.9 | 45.2 | 35.7 |
| **(5)** **Item 5:** Change of mind regarding telemedicine | 23.6 | 29.1 | 16.5 | 22.5 | 8.4 |

**Table S4. Spearman's rank correlation between questionnaire items (only patients with chronic illness**; **N = 454)**

|  | **(1)** | **(2)** | **(3)** | **(4)** | **(5)** |
| --- | --- | --- | --- | --- | --- |
| **(1)** **Item 1:** Necessity of using telemedicine during the COVID-19 crisis | 1 |  |  |  |  |
| **(2)** **Item 2:** Preference for going to a clinic during the COVID-19 crisis | -0.216** | 1 |  |  |  |
| **(3)** **Item 3:** Satisfaction with telemedicine services during the COVID-19 crisis | 0.229** | -0.226** | 1 |  |  |
| **(4)** **Item 4:** Willingness to use telemedicine in the future | 0.311** | -0.390** | 0.417** | 1 |  |
| **(5)** **Item 5:** Change of mind regarding telemedicine | 0.284** | 0.043 | 0.113* | 0.035 | 1 |

*Note:* *p < 0.05, **p < 0.001

**Ordered Logistic Regression Models**

**Predicting willingness to use telemedicine in the future**

Table S5 displays the different OLR models' results for predicting willingness to use telemedicine in the future. A model with only background variables (Model 1; log-likelihood = -456.92092, AIC = 947.842, BIC = 1025.039; McFadden Pseudo R^2^ =.043) was superior compared to a null model (i.e., model with no predictors; LR test: $\chi_{(15)}^{2}$ = 40.96, p<.000; log-likelihood = -376.1241, AIC = 958.8216, BIC = 967.9037). However, Model 2 with all possible predictors (that is, all the background variables and items 1 to 3) was superior compared with Model 1 (LR test: $\chi_{(12)}^{2}$= 173.04, p<.000; log-likelihood = -370.402, AIC = 798.803, BIC = 930.493; McFadden Pseudo R^2^ = .224). After using a forward-stepwise method, this model (log-likelihood of full model = -376.1241, AIC = 784.248, BIC = 856.905; McFadden Pseudo R^2^ =.212) was superior compared to Model 2 (LR test: $\chi_{(13)}^{2}$ = 11.44, p=.57). The final model is nested within the 'full' model (i.e., Model 2). The difference in BIC values between the two models and the insignificant LR test can indicate that the reduced model showed a better fit to the data than the 'full' model. Nonetheless, both models revealed very similar results concerning the associations between the different predictors and the dependent variable.

**Table S5. Different OLR models for predicting willingness to use telemedicine in the future**

| **Fit indices** | **Model 1** | **Model 2** | **Model 3** |
| --- | --- | --- | --- |
| **AIC** | 947.84 | 798.80 | 784.25 |
| **BIC** | 1025.04 | 930.49 | 856.91 |
| **McFadden Pseudo R^2^** | .04 | .22 | .21 |
| **Nagelkerke Pseudo R^2^** | .08 | .36 | .34 |
| **Model df** | 15 | 27 | 14 |
| *Note:* AIC = Akaike information criterion; BIC = Bayesian information criterion | | | |

**Predicting participants' change of mind regarding telemedicine**

Table S6 displays the different OLR models' results for predicting participants' change of mind regarding telemedicine. OLR test showed that a model with only background variables (Model 1a; log-likelihood = -652.283, AIC = 1396.313, BIC = 1473.511; McFadden Pseudo R^2^ = .022) was better compared to a null model (i.e., model with no predictors; LR test: $\chi_{(15)}^{2}$= 31.33, p<.01; log-likelihood = -696.820), although other fit indices were poorer (AIC = 1397.64, BIC = 1406.722). Model 2a with all possible predictors (that is all the background variables and items 1 to 3) was better compared to Model 1a (LR test : $\chi_{(12)}^{2}$ = 57.75, p<.000; log-likelihood = -652.283, AIC = 1362.566, BIC = 1494.256; McFadden Pseudo R^2^ = .064), and compared with a null model (LR test: $\chi_{(27)}^{2}$= 89.07, p<.000). After using a forward-stepwise method, Model 3a (log-likelihood of full model = -656.885, AIC = 1353.771, BIC = 1444.592; McFadden Pseudo R^2^ = .057) was superior compared to the null model and compared with model 1a (LR tests: $\chi_{(18)}^{2}$= 79.87, p<.000; $\chi_{(3)}^{2}$ = 48.54, p<.000; respectively). Model 3a did not differ significantly from Model 2a (LR test: $\chi_{(9)}^{2}$ = 9.21, p=.42), and was better in terms of AIC and BIC value. Both models revealed very similar results relating to the associations between the different predictors and the dependent variable.

**Table S6. Different OLR models for predicting participants' change of mind regarding telemedicine**

| **Fit indices** | **Model 1a** | **Model 2a** | **Model 3a** |
| --- | --- | --- | --- |
| **AIC** | 1396.313 | 1362.566 | 1353.771 |
| **BIC** | 1473.511 | 1494.256 | 1444.592 |
| **McFadden Pseudo R^2^** | .022 | .064 | .057 |
| **Nagelkerke Pseudo R^2^** | .051 | .139 | .126 |
| **Model df** | 17 | 29 | 20 |
| *Note:* AIC = Akaike information criterion; BIC = Bayesian information criterion | | | |
